# Shp2 Activates Fyn and Ras to Regulate RBL-2H3 Mast Cell Activation following FcεRI Aggregation

**DOI:** 10.1371/journal.pone.0040566

**Published:** 2012-07-10

**Authors:** Xiaoyun Fang, Yongjiang Lang, Yuxiong Wang, Wei Mo, Huanhuan Wei, Jianhui Xie, Min Yu

**Affiliations:** 1 The Key Laboratory of Molecular Medicine, Ministry of Education, Shanghai Medical College, Fudan University, Shanghai, People’s Republic of China; 2 Gene Research Center, Shanghai Medical College, Fudan University, Shanghai, People’s Republic of China; 3 Department of Biochemistry and Molecular Biology, Shanghai Medical College, Fudan University, Shanghai, People’s Republic of China; Indiana University School of Medicine, United States of America

## Abstract

The protein-tyrosine phosphatase (PTP) Shp2 has been implicated in many immunoreceptor signaling pathways, but its role in immunoreceptor FcεRI signaling, which leads to the activation of mast cells and blood basophils, is still largely undefined. Using Shp2 knockdown RBL-2H3 (RBL) mast cells, we here reported that Shp2 is required for the activation of RBL cells induced by FcεRI. FcεRΙ-evoked degranulation, calcium mobilization, and synthesis of cytokine transcripts (IL-1β, IL-10, and monocyte chemoattractant protein 1 (MCP-1)) were reduced in Shp2 knockdown RBL cells. Signaling regulatory mechanism investigation using immunoblotting, immunoprecipitation, and GST pull-down assay reveals that the down-regulation of Shp2 expression in RBL cells leads to decreased activities of Fyn, PLCγ, JNK, p38MAPK, and Ras/Erk1/2 after FcεRΙ aggregation. Further studies suggest that Paxillin phosphoryaltion was also impaired, but PAG phosphorylation was normal after FcεRΙ stimulation as a consequence of the inhibition of Shp2 expression in RBL cells. Collectively, our data strongly indicate that Shp2 is essential for the activation of RBL cells in response to FcεRΙ aggregation. Shp2 regulates this process through Fyn and Ras with no involvement of PAG. In addition, we identify Paxillin as an indirect substrate of Shp2 in FcεRΙ-initiated signaling of RBL cells.

## Introduction

The activation of mast cells via aggregation of the receptor FcεRI expressed on their surfaces is the central event in allergic diseases. Activated FcεRI with high-affinity for immunoglobulin E (IgE) contributes to inflammatory symptomology by inducing the release of proinflammatory mediators (degranulation), and the production of various cytokines and chemokines. Like many other immunoreceptors, FcεRΙ is made up of multiple chains, an IgE-binding section represented by the α subunit, and two signal transduction sections represented by the β and γ subunits, both of which contain ITAMs in tails [1], [2]. Upon the cross-linking of IgE-bearing FcεRΙ by polyvalent antigen, the β and γ subunits are phosphorylated at ITAMs by the receptor-proximal Src family protein tyrosine kinases (SFKs) [3], [4]. The Syk kinase is recruited to tyrosine-phosphorylated ITAMs and becomes activated to regulate its downstream targets in conjunction with the activated SFKs, such as Lyn, Fyn, and Hck [5]–[9]. A large number of downstream signaling relay molecules are involved in the following progress, including the linker for activated T cells (LAT) [10], phospholipase Cγ (PLCγ) [11], Grb2-associated binder 2 (Gab2) [12], [13], Phosphatidylinositol 3^'^-kinase (PI3K) [12], [14], Akt kinase and mitogen-activated protein kinases (MAPKs) [7], [12], [15]. Such tight assembly of these molecules together with the signal transduction among each other by means of phosphorylation and dephosphorylation eventually results in the activation of mast cells. This activation is characterized by enhanced calcium flux, prominent degranulation and increased gene expression activity [16], [17]. Two acknowledged signaling pathways initiated by SFKs, including Lyn/Syk/LAT [5], [6], [10], [18] and Fyn/PI3K [7], [12], [13], have been established to explain this process. However, the precise molecular mechanism involved in this progress is still not completely understood.

SFKs are important signaling relay molecules and the inhibitory C-terminal tyrosine (Tyr527 in mammalian Src) is their key regulatory site. When Tyr527 is phosphorylated by C-terminal Src kinase (Csk), the activities of SFKs are suppressed in resting-state. Csk is a cytoplasmic protein lacking a membrane targeting sequence. It can be recruited to the membrane where SFKs reside by binding of its SH2 domain to tyrosyl phosphorylated docking proteins, including transmembrane glycoprotein PAG/Cbp (PAG) and focal adhesion protein Paxillin, to exert its inhibitory effect on SFKs [19], [20]. This inhibitory state of SFKs can be rescued by the phosphorylation of Tyr416 (in mammalian Src), the activating regulatory site in SFKs [20]. The activation of SFKs is essential for mast cell activation in response to FcεRI engagement, but the signaling regulatory mechanism of SFK activation in FcεRI signaling remains elusive. Shp2 is an intracellular PTP and expressed ubiquitously. Increasing numbers of reports suggest that a wide range of biochemical functions are Shp2-dependent, such as embryonic development for mice and Xenopus [21], [22], Noonan syndrome, and Juvenile myelomonocytic leukemia [23]–[25]. Previous investigations have indicated that Shp2 is required for SFK activation in receptor-tyrosine kinase (RTK) signaling and integrin signaling [20], [26]. One widely accepted signaling mechanism involved in this regulation suggests that Shp2 dephosphorylates PAG and/or Paxillin to resist the recruitment of Csk to the membrane, leading to the activation of SFKs [20]. Another proposed explanation for this regulation supports the notion that Shp2 might directly dephosphorylate Tyr527, which results in Tyr416 activation via its loop autophosphorylation and therefore leads to SFK activation [27]. Recently, physical interaction of Fyn kinase with Shp2 in Madin-Darby canine kidney cells is reported, which may offer the possibility for such direct regulation [28]. In addition, Shp2 has been documented to be essential for Ras-MAPK/Erk1/2 activation in response to various growth factors and cytokines through RTK signaling [27]. Nevertheless, whether Shp2 has participations in FcεRΙ signaling to regulate the activation of SFK and Ras/Erk1/2 pathway remains to further be clarified.

Previous studies suggest a critical role of Shp2 in c-Kit signaling controlling proliferation and differentiation in mast cells [29], [30]. Furthermore, the involvement of Shp2 in FcεRΙ signaling has recently been reported [31]. However, little is known about how Shp2 regulates mast cell activation after FcεRΙ aggregation. In this study, we inhibited the expression of Shp2 in the rat mucosal mast cell analog, RBL cell line, with Shp2-specific shRNAs (Shp2 knockdown) or scramble shRNA (control) expressed by retroviruses to clarify the role of Shp2 in mast cell signaling through FcεRΙ.

## Results

### 1. Characterization of Shp2 Knockdown RBL Cells

**Figure 1:**
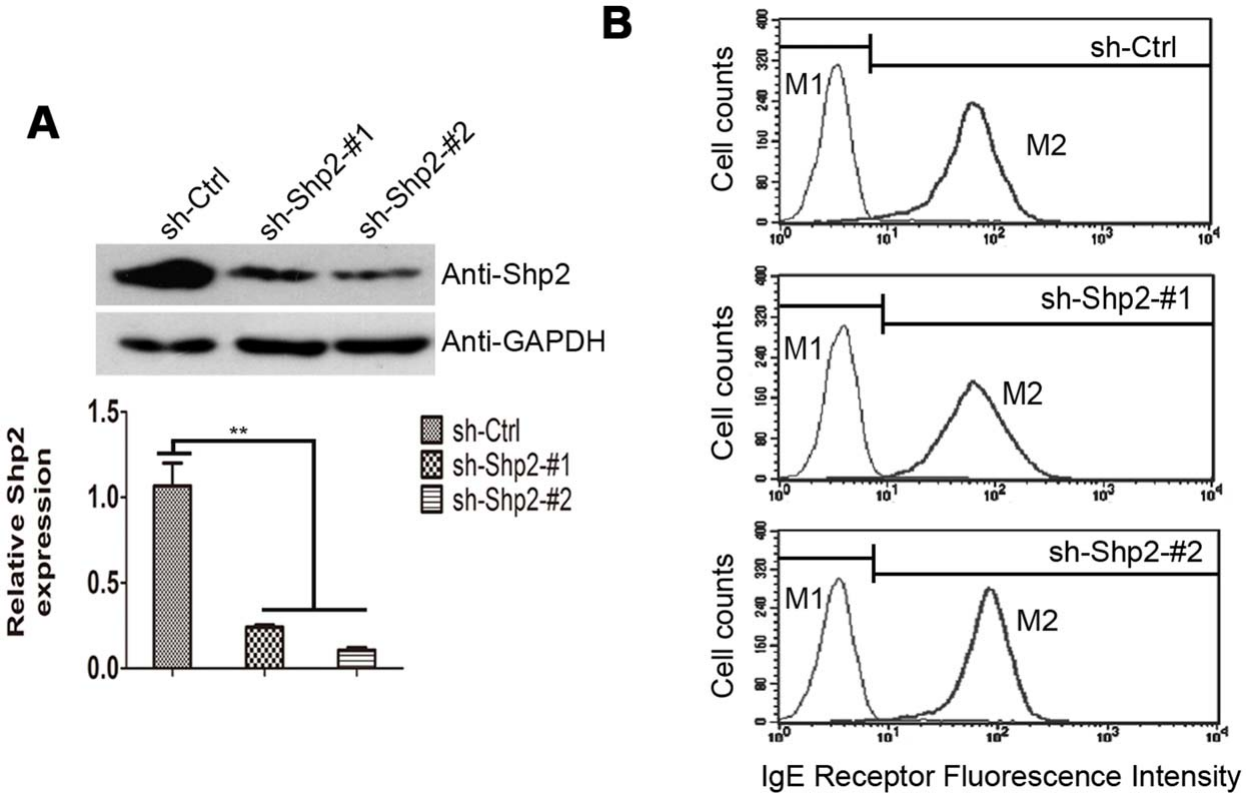
Effective inhibition of Shp2 expression in RBL cells using retroviral vectors expressing Shp2-sepcific shRNA. (A) Upper panels, WT RBL cells were infected with the viral supernatants containing recombinant viruses expressing Shp2-specific shRNAs (sh-Shp2-#1 and sh-Shp2-#2) or scramble shRNA (sh-Ctrl), respectively. Puromycin-resistant clones pooled after 5–7 days of infection were harvested to examine Shp2 expression by immunoblotting with anti-Shp2 antibody. Lower panel, Shp2 expression levels were quantified by densitometry in arbitrary units (AU) using ImageJ software, normalized to the GAPDH amount. Data are expressed as mean ± SEM of at least three experiments. **, *P*<0.01 by Student *t* test. (B) Surface expression of IgE receptor, FcεRΙ, is normal in Shp2 knockdown RBL cells. Three populations of RBL cells (sh-Ctrl, sh-Shp2-#1, and sh-Shp2-#2) were sensitized and analyzed for surface expression of FcεRΙ by FACS as described in Materials and Methods. Representative results of three experiments are shown.

To evaluate the role of Shp2 in FcεRΙ-induced activation of RBL cells, we established Shp2 knockdown RBL cell lines. WT RBL cells were transduced with retroviral vectors expressing shRNAs directed against Shp2 for knockdown effect (sh-Shp2-#1 and sh-Shp2-#2) or scramble shRNA for control (sh-Ctrl). Three populations of puromycin-resistant RBL cells (sh-Ctrl, sh-Shp2-#1, and sh-Shp2-#2) were collected and assayed for Shp2 expression by immunoblotting (Figure 1A). Expression of sh-Shp2-#1 and sh-Shp2-#2 cells led to reductions in Shp2 expression of 66.3% ±3.76%, and 88.2% ±0.35%, respectively, in RBL cells relative to scramble shRNA. On the contrary, expression of Shp1, homologous preotein of Shp2, was unchanged in Shp2 knockdown RBL cells (Figure S1). This indicates an effective and specific inhibition of Shp2 expression in RBL cells. Flow cytometry analysis showed that RBL cells from these three populations have similar surface expression of IgE receptor (Figure 1B), which suggests that the knockdown of Shp2 expression did not impair FcεRΙ expression.

### 2. Shp2 is Required for FcεRΙ-Mediated RBL Cell Activation

**Figure 2:**
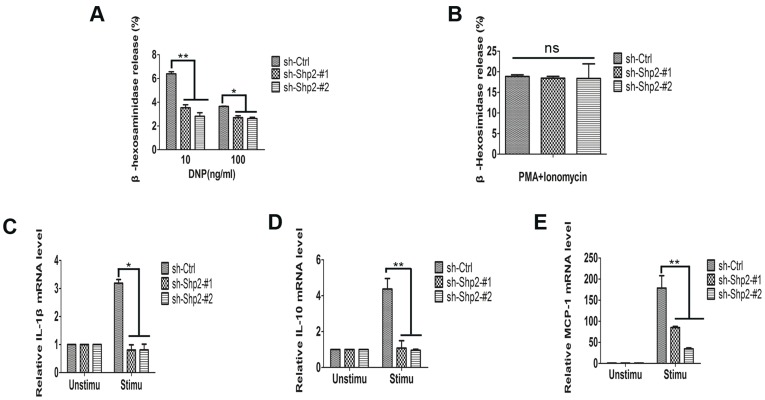
Shp2 is required for FcεRΙ-evoked degranulation and synthesis of cytokine mRNAs in RBL cells. (A–B) FcεRΙ-evoked degranulation is defective in Shp2 knockdown RBL cells. Three populations of RBL cells sensitized by anti-DNP-IgE were starved and stimulated with 10 or 100 ng/ml DNP (A) or PMA plus ionomycin (B). The levels of degranulation were determined by measuring the release of β-hexosaminidase. Results are reported as the percentage of total net release. Representative data expressed as mean ± SEM from one of at least 3 experiments are shown. **, *P*<0.01; *, *P*<0.05. ns, not significant as determined by Student *t* test compared to controls. (C-E) FcεRΙ-induced generation of cytokine mRNAs is significantly inhibited in Shp2 knockdown RBL cells. Three populations of RBL cells were sensitized, starved, and stimulated with 10 ng/ml DNP for 1 h, or left unstimulated. Total RNA was isolated and synthesis of IL-1β (C), IL-10 (D), and MCP-1 (E) transcripts was detected by real-time RT-PCR. Values were normalized to GAPDH expression and presented as mean ± SEM. Representative results from one of a minimum of 4 experiments are shown. **, *P*<0.01, Student *t* test.

To assess the functional role of Shp2 in RBL cell degranulation and cytokine production after FcεRΙ aggregation, we investigated the consequences of the knockdown of Shp2 expression. Three populations of RBL cells (sh-Ctrl, sh-Shp2-#1, and sh-Shp2-#2) were stimulated with different concentrations of the antigen DNP-BSA (DNP) and degranulation was determined by the release of β-hexosaminidase (Figure 2A). We observed that degranulation levels in Shp2 knockdown RBL cells (3.54% ±0.44% for sh-Shp2-#1, and 2.83% ±0.50% for sh-Shp2-#2) were significantly lower than in controls (6.39% ±0.31%; values correspond to 10 ng/ml DNP stimulation, the concentration of optical stimulation). In contrast, degranulation induced by 12-O-tetradecanoylphorbol-13-acetate (PMA) plus ionomycin remained at a parallel level among three populations of RBL cells (Figure 2B). This implies that the secretion machinery is normal in Shp2 knockdown RBL cells and the defective degranulation is attributable to the inhibition of Shp2 expression. Besides, reductions in the degranulation in sh-Shp2-#2 population were more pronounced than in sh-Shp2-#1 population, which corresponded to the extent of the inhibition of Shp2 expression revealed by immunoblotting in Figure 1A. Transcripts of cytokines were subsequently determined by real-time RT-PCR. A significant decrease of the generation of IL-1β (Figure 2C), IL-10 (Figure 2D), and MCP-1 (Figure 2E) transcripts after FcεRΙ stimulation was observed in Shp2 knockdown RBL cells compared with control cells.

**Figure 3:**
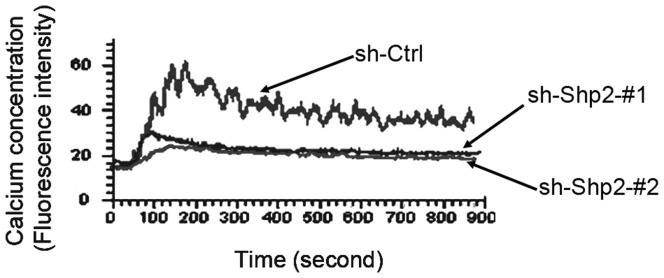
Shp2 is required for FcεRΙ-evoked calcium mobilization in RBL cells. Three populations of RBL cells were sensitized with anti-DNP IgE and loaded with Fluo3-AM. The fluorescence intensity after 10 ng/ml DNP stimulation was continuously monitored with a laser scanning confocal microscope. Results presented by the mean values of more than 20 cells in every experiment are shown. Each panel is representative of two independent experiments.

Given that mast cell degranulation is calcium-dependent, we examined the contribution of Shp2 to FcεRΙ-evoked calcium mobilization. The calcium flux of RBL cells from the three populations was monitored over time following DNP stimulation (Figure 3). Similar to degranulation, calcium response was markedly inhibited in Shp2 knockdown RBL cells compared with control cells. Collectively, our data strongly indicate that Shp2 is required for FcεRΙ-mediated RBL cell activation.

### 3. SFK Activation is Impaired in Shp2 Knockdown RBL Cells

**Figure 4:**
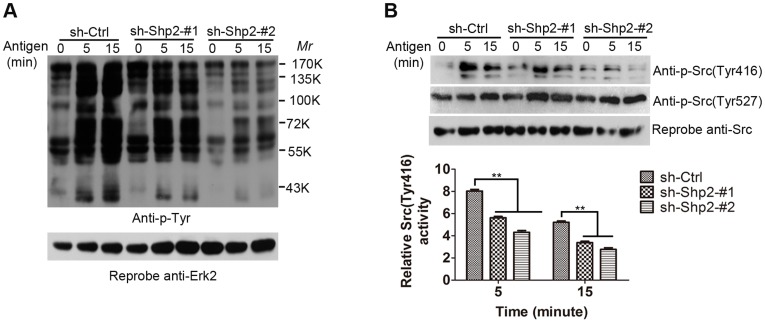
Shp2 knockdown results in defective phosphorylation of intracellular signaling relay molecules. (A) Shp2 knockdown RBL cells show globally defective of tyrosyl phosphorylation. Sensitized RBL cells from the three populations of RBL cells were starved and stimulated with 10 ng/ml DNP for the indicated times. WCL were immunoblotted with anti-phosphotyrosine (p-Tyr, 4G10) antibody (A, upper lane). The membrane was stripped and reprobed with anti-Erk2 antibody to control for loading (A, lower lane). Representative blots of more than three experiments are shown. (B) Shp2 knockdwon RBL cells show defective activation of SFK. Upper panels, WCL got as A were subjected to immunoblotting with anti-p-Src (Tyr416 and Tyr527) antibodies. The blots were stripped and reprobed with anti-Src antibody for loading control. Lower panel, Src (Tyr416) phosphorylation is qualified by densitometry in AU and normalized to total amount of Src. Data are expressed as mean ± SEM and representative results of at least three experiments are shown. **, *P*<0.01, Student *t* test.

We next explored the FcεRΙ-evoked signaling events caused by the inhibition of Shp2 expression. To evaluate the global effect of Shp2 knockdown on FcεRΙ signaling, we determined the overall level of intracellular tyrosyl phosphorylation by immunoblotting (Figure 4A). A marked reduction in the amount of tyrosine-phosphorylated proteins was observed in the lysates from FcεRΙ-stimulated Shp2 knockdown RBL cells compared with control cells, which accounts for the positive role of Shp2 in FcεRΙ-evoked RBL cell activation. For a more specified molecular mechanism, lysates from the three activated populations of RBL cells were subjected to immunoblotting with antibodies against the phospho (p)-Src (activating site Try416 and inhibitory site Tyr527). As showed in Figure 4, the phosphorylation of Tyr416 required for mast cell activation was largely inhibited in Shp2 knockdown RBL cells compared with control cells. In contrast, Tyr527 phosphorylation was even slightly enhanced in Shp2 knockdown RBL cells. The data suggest that Shp2 has a role in regulating FcεRΙ-evoked SFK activation in RBL cells and the inhibitory C-terminal tyrosine in SFKs seems to be one of potential target sites for Shp2.

### 4. Fyn Kinase Activation is Defective in Shp2 Knockdown RBL Cells

**Figure 5:**
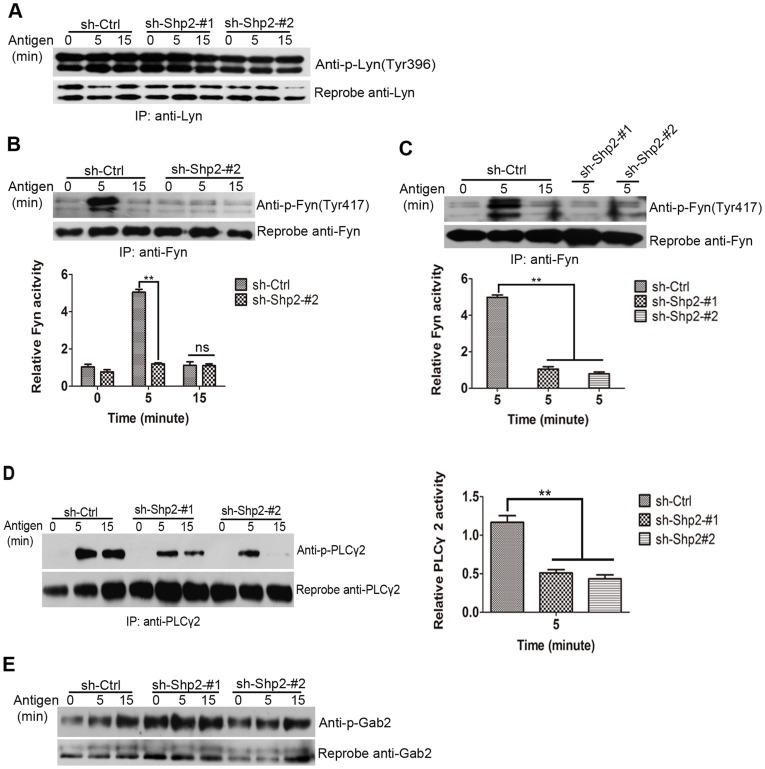
Shp2 regulates the activity of Fyn kinase, but not Lyn kinase. (A) Three populations of RBL cells were stimulated with 10 ng/ml DNP for the indicated times. Lyn was immunoprecipitated and amounts of phosphotyrosine of activating site were determined by immunoblotting. To control for loading, blots were stripped and reprobed with anti-total Lyn antibody. Representative blots of at least 3 experiments are shown. (B–C) Upper panels, lysates from the indicated populations of RBL cells activated by receptor aggregation were immunoprecipitated with Fyn followed by immunoblotting as in A. Representative blots of at least three experiments are shown. Lower panels, Fyn phosphorylation is qualified by densitometry in AU and normalized to total Fyn. (D) Left panels, lysates from three populations of RBL cells after stimulation were immunoprecipitated with PLCγ2 antibody followed by immunoblotting as in A. Right panels, PLCγ2 activity is qualified by densitometry in AU and normalized to total amount of PLCγ2. Representative blots of at least three experiments are shown. (E) WCL from three populations of RBL cells after stimulation were immunoblotted with anti-p-Gab2 antibody. Blots were stripped and reprobed with anti-total Gab2 antibody to control for loading. Representative blots of at least three experiments are shown. For all experiments, data are expressed as mean ± SEM and representative results of at least 3 experiments are shown. **, *P*<0.01. ns, not significant compared to controls, as determined by Student *t* test.

Among SFKs, Fyn and Lyn kinases are two important members for mast cell activation. To determine whether the activation of Lyn and Fyn is Shp2-dependent, we analyzed the kinetics of their phosphorylation after FcεRΙ aggregation in the three populations of RBL cells by immunoprecipitation with Lyn (Figure 5A) and Fyn (Figure 5B–C). Similar levels of Lyn tyrosyl phosphorylation (Tyr396, the activating site) were detected in the three populations. On the contrary, Fyn activation was remarkably decreased in Shp2 knockdown populations compared with control population. The control population showed robust tyrosyl phosphorylation (Tyr417, the activating site) of Fyn kinase, which peaked at 5 min and decreased at 15 min after stimulation. However, only weak phosphorylation signals were detected after FcεRΙ aggregation in Shp2 knockdown populations, even at the time of peak stimulation. Furthermore, the phosphorylation of both PLCγ1 (Figure S2) and PLCγ2 (Figure 5D), two signaling relay molecules downstream of Fyn, was also defective in Shp2 knockdown populations. Of note, knockdown of Shp2 expression did not affect the expression of Lyn, Fyn, or PLCγ proteins. Therefore, our results demonstrate that Shp2 regulates FcεRΙ-evoked RBL cell activation via Fyn kinase, but not Lyn kinase.

Additionally, Gab2 tyrosyl phosphorylation was hardly affected in Shp2 knockdown populations compared with control population (Figure 5E). This indicates that Gab2 phosphorylation is almost Fyn-independent in response to FcεRΙ stimulation in RBL cells.

### 5. Shp2 Regulates Fyn Activation Independent of PAG

**Figure 6:**
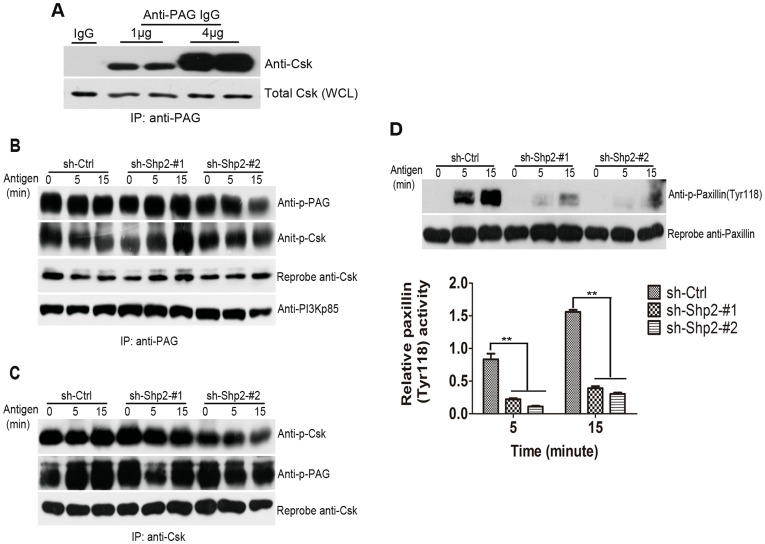
Shp2 knockdown results in unchanged phosphorylation of PAG, but imparied phosphorylation of Paxillin. (A) PAG coimmunoprecipitates with Csk. Cell lysates from the resting WT RBL cells were immunoprecipitated with varying amounts of mouse anti-PAG IgG (1 µg and 4 µg, respectively) or mouse isotype IgG (IgG) followed by immunoblotting with anti-Csk antibody. The WCL were subjected to immunoblotting with anti-Csk antibody to control for total Csk. Representative blots of two experiments are shown. (B–C) Shp2 knockdown RBL cells show normal association of PAG with Csk. Three populations of RBL cells were sensitized, starved and stimulated with 10 ng/ml DNP for the indicated times. (B) PAG was immunoprecipitated and phosphotyrosine contents were determined by immunoblotting with anti-p-Tyr antibody (4G10). To examine the amounts of Csk associated with PAG, the blots were stripped and reprobed with anti-Csk antibody. Representative blots of at least three experiments are shown. (C) Csk immunoprecipitates were determined for phosphorylation as in B. Blots were stripped and reprobed with anti-Csk antibody for loading control. Representative blots of at least three experiments were shown. (D) Upper panels, Shp2 knockdown RBL cells show reduced phosphorylation levels of Paxillin. Three populations of RBL cells were stimulated as in B. WCL were immunoblotted with anti-p-Paxillin (Tyr118) and Paxillin antibodies. Lower panel, Paxillin phosphorylation is qualified by densimentry in AU and normalized to total amount of Paxillin. Data are expressed as mean ± SEM and representative results from at least three experiments are shown. **, *P*<0.01, Student *t* test.

The inhibition of Shp2 expression resulted in markedly diminished Fyn activation after FcεRΙ aggregation (Figure 5B–C). This implicates that Shp2 can promote dephosphorylation of the inhibitory C-terminal of Fyn in some way. To determine whether Shp2 could dephosphorylate PAG to decrease the association of Csk and thus promote Fyn activation after FcεRΙ engagement, we monitored the dynamic phosphorylation of PAG following antigen stimulation in three populations of RBL cells. The association of Csk with PAG in quiescent WT RBL cells was first confirmed (Figure 6A), which is consistent with the previous report [32]. Next, immunoprecipitation was performed on lysates from the three activated populations of RBL cells. Immunoprecipitation of PAG followed by immunoblotting with the antibody against phosphotyrosine (4G10) showed that the levels of PAG tyrosyl phosphorylation (Figure 6B, upper lane), and its association with Csk (Figure 6B, third lane), in Shp2 knockdown populations were equivalent to that in control population. Similarly, Csk immunoprecipitates displayed comparable overall intensity of tyrosyl phosphorlation of both PAG and Csk (Figure 6C). These data clearly demonstrate that PAG is not involved in the regulation of Shp2-dependent Fyn activation in FcεRΙ signaling. When we analyzed the phosphorylation of Paxillin, another Csk-binding protein and potential substrate of Shp2, we found that the tyrosyl phosphorylation levels of Paxillin were elevated at 5 min of stimulation, and markedly enhanced at 15 min after stimulation in control population, whereas Shp2 knockdown populations showed significantly inhibited Paxillin tyrosyl phosphorylation after stimulation (Figure 6D). This indicates that Shp2 promotes, rather than inhibits, Paxillin phosphorylation in FcεRΙ signaling.

### 6. Ras and MAPKs Activities are Inhibited in Shp2 Knockdown RBL Cells

**Figure 7:**
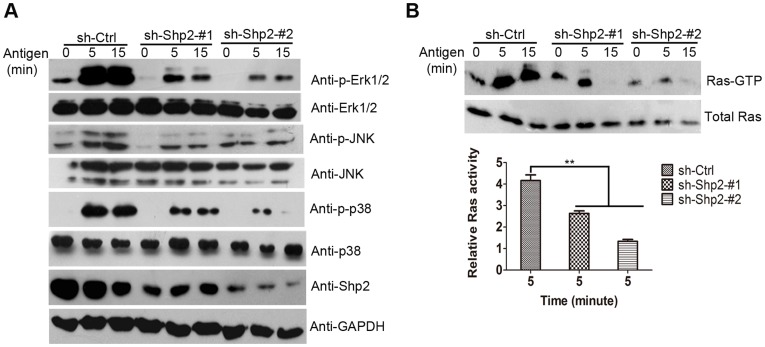
Shp2 knockdown results in defective activation of Ras and MAPKs. (A) Sensitized RBL cells from the three populations were starved and stimulated with 10 ng/ml DNP for the indicated times. WCL were immunoblotted with anti-p-Erk1/2 and anti-p-JNK and anti-p-p38 antibodies to measure the phosphorylation levels of MAPKs. To control for loading, blots were stripped and reprobed with anti-total Erk1/2, JNK, and p38 antibodies. Shp2 expression was also determined by immunoblotting with anti-Shp2 antibody followed by reprobing with anti-GAPDH antibody. Representative blots of at least 3 experiments are shown. (B) Upper panels, RBL cells from the three populations were sensitized, starved, and stimulated with 10 ng/ml DNP for indicated times and WCL were prepared. Active Ras (Ras-GTP) amounts in WCL were determined by incubating the WCL with GST-RBD, followed by immunoblotting with anti-Ras antibody according to Materials and Methods. 5–10% of WCL of each sample was loaded as a control for the total Ras in RBL cells. Lower panel, activated Ras was measured by quantifying the Ras-GTP levels at 5 min after stimulation normalized to total Ras. Data are expressed as mean ± SEM and representative results from at least three experiments are shown. **, *P*<0.01, Student *t* test.

The activation of MAPKs is a remarkable event after FcεRΙ aggregation responsible for production of many types of cytokines [33], [34]. To understand whether Shp2 has a role in FcεRΙ-evoked MAPK activation, we determined the phosphorylation of MAPKs, including Erk1/2, JNK, and p38, in the three populations of RBL cells by immunoblotting (Figure 7A). As indicated by a marked decrease in the phosphorylation levels of MAPKs in Shp2 knockdown populations compared with control population, our data suggest that Shp2 is required for optimal MAPK activation in response to FcεRΙ aggregation. To investigate the relationship between Shp2 and Ras, an upstream molecule of Erk1/2, in FcεRΙ signaling, GST-RBD (Ras binding domain in Raf) pull down assay was carried out to compare the amount of active Ras (in the form of GTP-bound) after FcεRΙ stimulation in the three populations of RBL cells (Figure 7B). The amount of Ras-GTP at 5 min after stimulation was reduced by 40–70% in Shp2 knockdown populations compared with that in control population. Therefore, our results suggest that Shp2 positively regulates Ras/Erk1/2 signaling as well as the activation of JNK and p38MAPK in response to FcεRΙ stimulation in RBL cells.

## Discussion

In this work, we studied the role of Shp2 in FcεRΙ signaling utilizing Shp2-specific shRNAs expressed by retroviruses to inhibit Shp2 expression in RBL cells. Although previous reports have demonstrated a crucial contribution of Shp2 in development [35], proliferation [29], [36]–[38], and tumor suppression [39]–[41], little is known about the role of Shp2 in mast cell-dependent allergic reactions. Our results demonstrate an essential role of Shp2 in FcεRI-mediated RBL cell activation. This conclusion is based on observation of inhibited degranulation (Figure 2A), synthesis of cytokine mRNAs (IL-1β, IL-10, and MCP-1; Figure 2C–E), and calcium flux (Figure 3) in Shp2 knockdown RBL cells after FcεRI aggregation. Furthermore, our examination reveals that Shp2 could activate Fyn and Ras to promote FcεRI-evoked activation of RBL cells. These results contribute to our understanding of the signaling regulatory mechanism involved in the activation of RBL cells after FcεRΙ aggregation and raise the possibility of targeting Shp2 as a new therapeutic strategy for inflammatory diseases.

In mast cells, FcεRΙ aggregation activates a complicate signal transduction cascade, the major pathway of which is Lyn/Syk/LAT signaling. In the absence of either Syk or LAT, mast cells show impaired degranulation, cytokine generation, and PLCγ phoshprylation in response to FcεRΙ stimulation [5], [10]. These defects are similar to those in the inhibition of Shp2 expression (Figure 2, 5D). This conformity prompted us to examine the activity of Lyn kinase in Shp2 knockdown RBL cells after FcεRΙ cross-linking. Unexpectedly, Lyn kept showed continued high phosphorylation levels after antigen stimulation in both control and Shp2 knockdown RBL cells, without significant difference between the two (Figure 5A). Furthermore, the synthesis of MCP-1 mRNA induced by FcεRΙ aggregation was markedly inhibited in both Shp2 knockdown RBL cells (Figure 2E) and Fyn-deficient BMMCs [42], but was unaffected in LAT-deficient BMMCs [10]. These data indicate that Shp2 is probably involved in Fyn-dependent signaling cascade, which is independent of Lyn but has some cross-talk with Syk/LAT signaling. Taking that Fyn is essential for the optimal activation of Syk after FcεRI engagement into consideration [13], Fyn/PI3K signaling, the pathway initiated by Fyn kinase is an attractive candidate for such regulation [12], [13]. In fact, FcεRΙ-induced Fyn activation was largely diminished on account of the inhibition of Shp2 expression in RBL cells (Figure 5B–C). Moreover, FcεRI-evoked degranulation and phosphorylation of PLCγ, JNK, and p38MAPK were all reduced in Shp2 knockdown RBL cells (Figure 2A, 5D, and 7A), similar to that reported in Fyn-deficient BMMCs [7], [13], [42]. We thus propose that Shp2 promotes FcεRI-evoked RBL cell activation via Fyn/PI3K/PLCγ signaling, in which Syk may function as a mediator in the transmission of signals from FcεRΙ/Shp2/Fyn to PI3K. PI3K is important for the phosphorylation of PLCγ, which is able to cleave phosphatidylinositol-4,5-bisphosphate (PIP2) into inositol 1,4,5-trisphosphate (IP3) and diacylglycerol (DAG), leading to the elevation of intracellular calcium concentration with a contribution to degranulation [12], [43]. In this way, our proposed regulatory model of Shp2 explains the defective degranulation (Figure 2A) and calcium mobilization (Figure 3).

The fact that Gab2 phosphorylation was largely unaffected in Shp2 knockdown RBL cells (Figure 5E) indicates a loose interaction of Gab2 with Fyn in FcεRΙ signaling. This is consistent with our previous observation that FcεRΙ-mediated phosphorylation of Gab2 was severely impaired in Lyn-deficient BMMCs, but almost normal in Fyn-deficient BMMCs [13], which suggests a requirement of Lyn to a greater extent than that of Fyn for Gab2 phosphorylation in FcεRΙ signaling. In agreement with this suggestion, FcεRΙ-evoked phosphorylation of both Lyn and Gab2 was hardly affected in Shp2 knockdown RBL cells (Figure 5A and 5E). Nevertheless, more experiments are necessary to be carried out to delineate the regulatory signaling mechanism of Gab2 phosphorylaiton in response to FcεRΙ activation. The most important function for a PTP is to dephosphorylate its specific targets leading to inactivation of its targets [44]. Given that Shp2, as a PTP, functions positively in FcεRΙ-evoked RBL cell activation, we assumed that, as reported in RTK signaling [20], the phosphorylation levels of Shp2 substrates: PAG and Paxillin would elevate after FcεRΙ stimulation in Shp2 knockdown RBL cells compared with control cells. However, as our results show, although Shp2 expression was inhibited, the phosphorlation levels of PAG after FcεRΙ stimulation were not increased compared with that of control cells (Figure 6B, upper lane); nor was enhanced association of PAG with Csk observed (Figure 6B, third lane). This suggests that Shp2-mediated signaling downstream of the FcεRΙ might require no involvement of PAG. Phosphorylation levels of Paxillin were decreased, but not elevated in Shp2 knockdown RBL cells (Figure 6D), indicating that Paxillin lays downstream of Shp2 acting as an indirect, rather than a direct substrate of Shp2 in FcεRΙ signaling. This aids us to exclude the possibility that Shp2 regulates FcεRΙ-evoked Fyn activation by dephosphorylating Paxillin to reduce the binding of Csk. Based on these results, it is possible that Shp2 promotes FcεRΙ-induced Fyn activation in RBL cells by directly dephosphorylating the inhibitory C-terminal of Fyn kinase. Further investigations are required to confirm this speculation. Besides, reduced Paxillin tyrosyl phosphorylation due to the inhibition of Shp2 expression in FcεRI signaling is similar to that in integrin signaling. The marked response of Paxillin phosphorylation delayed at 15 min after DNP stimulation was also consistent with that in response to integrin stimulation [26]. These findings have implications for considering the potential role of Shp2 in contributing to RBL mast cell adhesion and migration, which are of great importance in allergic reactions.

It should be noted that both Lyn and PAG phosphorylation were not significantly enhanced in response to antigen sitmulaiton in our results. However, it does not mean that their phosphorylation cannot be induced in FcεRΙ signaling. In fact, it has been documented that Lyn phosphorylation is elevated after FcεRΙ aggregation. Once antigen stimulation, the aggregated FcεRΙ is supposed to associate with more Lyn kinases and activate them, thereby amplifying signals. But as the most upstream molecule in FcεRΙ signaling, Lyn completed this process rapidly, always less than 2 min [32]. We guess that our time for stimulation may be too long (5 min and 15 min) to capture the robust change of Lyn phosphorylation, leading no difference observed before and after stimulation in our results. Anyway, given that Lyn is constitutively associated with the receptor even in quiescent RBL cells, Lyn phosphorylation would be affected even in unstimulated mast cells if Shp2 has effects on Lyn activation, i.e. Shp2 knockdown RBL mast cells should show different phosphorylation levels from that of controls. Therefore, our stimulation times did not prevent us from assessing the role of Shp2 in Lyn activation. Similarly, it is also applies for PAG phosphorylation.

In addition, it seems that Shp2 is required for the activation of MAPKs after FcεRΙ engagement in RBL cells (Figure 7A). Defective gene expression in Shp2 knockdown RBL cells (Figure 2C–E) further supports its essential role in FcεRΙ-induced MAPK activation [33]. Impaired Fyn activation may account for the reduced activities of JNK and p38MAPK [42], whereas largely decreased Erk1/2 activity is due to the defective activation of Ras [27], [45], [46]. Importantly, reduced Ras activity in Shp2 knockdown RBL cells (Figure 7B) facilitates us to place Shp2 upstream of Ras in FcεRΙ signaling. This suggests a positive role of Shp2 in Ras/Erk1/2 signaling downstream of the IgE receptor in RBL cells, which is consistent with previous studies conducted in other cells or receptor signaling (e.g. RTK signaling) [27], [36]. However, when we focused on the question how Shp2 causes the activation of Ras, we found that there exists a matter of debate. The involved mechanisms are often different across cases, depending on cell types and/or stimulation factors [46]. As a common explanation in this community, Shp2 is supposed to dephosphorylate RasGAP-binding sites on receptors [47] or sprouty proteins [48], [49], which negatively regulates Ras activation. As we did not detect sprouty2 expression in RBL cells, it may be an attractive field to further explore whether it has RasGAP-binding sites on FcεRΙ and whether Shp2 could promote Ras activation by dephosphorylating these sites in mast cells.

**Figure 8:**
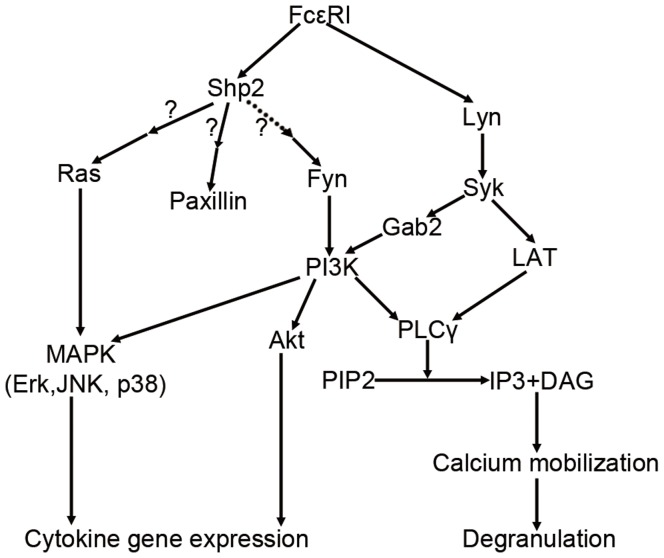
Model of Shp2 action in FcεRΙ signaling. Upon FcεRΙ stimulation, Fyn kinase and Ras become activated in a Shp2-dependent manner. Activated Fyn transmits positive signals to PI3K, leading to PI3K activation that accounts for calcium mobilization as well as for Akt phosphoryaltion. MAPKs also become phosphorylated following Ras and Fyn activation that are responsible for production of most cytokines. Paxillin is phosphoryated indirectly by Shp2 upon FcεRΙ stimulation. The direct substrates of Shp2 in FcεRΙ signaling remain to be defined. Another acknowledged signaling pathway Lyn/Syk/LAT is also shown.

We did not determine the relationship between Fyn and Ras, or Fyn and Paxillin. More efforts must be made to clarify the relationships among these signaling relay molecules. Actually, there have been no reports demonstrating a direct interaction between Fyn and Ras or Paxillin to date. Our study therefore suggests that Shp2 regulates FcεRΙ-evoked RBL cell activation via Fyn kinase and Ras/Erk1/2 signaling (Figure 8).

## Materials and Methods

### 1. Antibodies and Reagents

Rabbit antibodies to Shp2, Lyn, Ras, Erk1/2, p38, JNK, Akt, Src, PLCγ1, phospho-Src (Tyr416 and 527), phospho-Paxillin (Tyr118), phospho-Erk1/2, phospho-JNK, phospho-p38 and phospho-Akt (Ser473) were from Cell Signaling Technology (Danvers, MA, USA). Rabbit antibody to Erk2, PLCγ2, Shp1, and HRP-conjugated antibodies to mouse and rabbit immunoglobulin were from Santa Cruz Biotechnology (Santa Cruz, CA, USA). Mouse monoclonal antibodies to Fyn and Csk-binding protein (PAG) were from Pierce Biotechnology (Rockford, IL, USA). Mouse monoclonal antibody to Csk and Paxillin were from BD Biosciences (San Jose, CA, USA). Mouse monoclonal antibody to phosphotyrosine (4G10) was from Millipore (Bedford, Mass, USA). Monoclonal anti-dinitrophenyl (DNP)-IgE and DNP-bovine serum albumin (BSA) were from Sigma-Aldrich (St. Louis, MO, USA). FITC-conjugated monoclonal rat anti–mouse IgE (IgG1) and rat monoclonal IgG1were from from BD Pharmingen (San Jose, CA**,** USA). Leupeptin, aprotinin, pepstatin, antipain, β-glycerophosphate, sodium fluoride (NaF), Sodium orthovanadate (Na_3_VO_4_), and phenylmethylsulfonylfluoride (PMSF) were from Sigma-Aldrich.

### 2. Cell Lines and Culture

RBL-2H3 cell lines [50] and ecotrophic packaging line Plate-E [51]were cultured in MEM (Gibco) and DMEM (Gibco), respectively, with 10% heat-inactivated FBS (HyClone), 2 mM glutamine (Gibco), 100 µg/ml streptomycin (Gibco) and 100 units/ml penicillin (Gibco) in a humidified incubator filled with 5% CO_2_ at 37°C. For sensitization, cells were incubated with anti-DNP-IgE (0.1 µg/ml) overnight.

### 3. Plasmid Constructs

shRNA constructs were kind gifts from Dr. Richard Chan of Ontario Cancer Institute’s Cancer Biology Progra (Toronto, Ontario, Canada). Two 21-nucleotide sequences are as follows:

sh-Shp2-#1∶5'-AAGATTCAGAACACTGGGGAC-3'

sh-Shp2-#2∶5'-AAGAGTATGGCGTCATGCGTG-3'

### 4. Retroviral Infection of RBL Cells

pSuper-puro retroviral plasmids were transfected into Plate-E cells using Lipofectamine 2000 (Invitrogen, Carlsbad, CA,USA). Viral particle-containing culture supernatants were collected after 24, 36, and 48 h, respectively. The supernatants were filtered through 0.45 µm filters and the viral supernatant was used to infect RBL cells after addition of 4 µg/ml polybrene (Sigma-Aldrich). Infected RBL cells were then incubated at 37°C for 12 h. After 12 h, the infected cells were selected in the presence of 3 µg/ml puromycin for 5–7 days. Mass populations of puromycin-resistant RBL cells (sh-Ctrl, sh-Shp2-#1, and sh-Shp-#2) were used for experiments.

### 5. Flow Cytometry Analysis (FACS)

Sensitized RBL cells were stained for FcεRΙ expression with FITC-rat-anti-mouse IgE monoclonal antibody (IgG1) or FITC-isotype control (rat monoclonal IgG1) antibody for 1 h on ice, followed by flow cytometry analysis using a FACScan (Becton Dickinson FACStar).

### 6. Immunoprecipitation and Immunoblotting

Sensitized RBL cells were starved in MEM containing 2% BSA (Amersco, Solon, Ohio, USA) overnight, and either stimulated with DNP (10 ng/ml) or left unstimulated for the indicated times. Cells were lysed for 40 min at 4°C with NP-40 lysis buffer (50 mM Tris-HCl [pH 7.5], 150 mM NaCl, 0.1% NP-40, 5 mM EDTA, 5 mM EGTA, 15 mM MgCl_2_, 60 mM β-glycerophosphate, 10 mM NaF, 2 mM Na3VO4, 200 mM PMSF, 4 µg/ml aprotinin, 20 µg/ml leupeptin, 2 µg/ml antipain, and 2 µg/ml pepstatin). The lysates (1 mg of total protein) were precleared by incubating together with 20 µl of protein G-Sepharose (Roche Applied Science, Mannheim, Germany) for 3 h at 4°C. Precleared lysates were centrifuged and transferred into new tubes followed by incubating with the indicated antibodies and 30 µl of protein G-sepharose overnight at 4°C. Immunoprecipitates were resolved by SDS-PAGE, transferred to polyvinylidene difluoride (PVDF) membranes (Roche Applied Science), and probed with the appropriate primary antibodies followed by secondary goat anti-mouse or anti-rabbit antibodies. For immunoblotting, whole cell lysates (WCL) were mixed with 5 × sample buffer after determination of protein concentrations using a bicinchoninic acid (BCA) protein assay kit (Pierce Biotechnology). They were fractionated onto SDS-PAGE as described above.

### 7. GST-Pull Down Assay

Ras-GTP pull down assay was performed as described [52]. Briefly, the GST-RBD (the Ras binding domain in Raf) fusion protein was produced in *Escherichia coli* DH5α-competent bacteria and purified by glutathione agarose beads (Sigma-Aldrich) on the day of the assay. Cells were lysed with NP-40 buffer as described in the Immunoprecipitation and Immunoblotting section. The lysates were incubated with GST-RBD at 4°C for 1 h. The complex was washed, resolved using SDS-PAGE and immunoblotted with Ras antibody.

### 8. β-hexosaminidase Assay

Degranulation assay was performed as described [53] with the following modifications. RBL cells were stimulated with 100 µl of DNP or PMA plus ionomycin, or left unstimulated for 1 h at 37°C. 100 µl of specific substrate for β-hexosaminidase 4-methylumbelliferyl-*N*-acetyl-β-D-glucosaminide (2.4 mM, Sigma-Aldrich) was added and incubated for another 30 min at 37°C. Aliquots (50–100 µl) of the supernatant were transferred into a new 96-well tissue culture plate and analyzed using a fluorescence microplate reader (Tecan Inc.) as described [53].

### 9. Measurement of Calcium Flux

Sensitized RBL cells were stained with 4 µM Fluo3-AM (Dojindo, Japan) in Tyrode’s buffer (135 mM NaCl, 5 mM KCl, 1.8 mM CaCl_2_, 1.0 mM MgCl_2_, 5.6 mM glucose, 20 mM HEPES, and 1 mg/mL BSA at pH 7.4) for at least 45 min at 37°C. The stained cells were challenged with DNP (10 ng/ml) for the indicated lengths of time. Single wavelengths was collected at 520 nm (488 nm excitation) over time and monitored using a laser scanning confocal microscope (Leica Microsystems Heidelberg GmbH, Germany). Changes in [Ca^2+^] were reflected by varied fluorescence intensity of Ca^2+^-bound Fluo-3. Calcium flux-time curves were captured and analyzed using the “LAS AF Lite” software package (Leica), and the values were measured after subtracting the background fluorescence.

### 10. Real-Time RT-PCR

Sensitized RBL cells were stimulated with DNP (10 ng/ml), or left unstimulated, in Tyrode’s buffer for 1 h at 37°C. Total RNA was isolated using the TRI reagent (Invitrogen). First-strand complementary DNA (cDNA) was obtained as described [10]. The synthesized cDNA was subjected to real-time RT-PCR conducted by an ANTIBODYI 7300 real-time PCR instrument (Applied Biosystems) using a SYBR green-based real-time PCR kit (Takala, Japan) according to the manufacture’s instruction. The primers used were as follows:

MCP-1∶5′-ATGCAGTTAATGCCCCACTC-3′(sense), 5′-TTCCTTATTGGGGTCAGCAC-3′ (antisense);

IL-1β: 5′-CACAGCAGCATCTCGACAAGA-3′ (sense),

5′-CACGGGCAAGACATAGGTAGCT-3′ (antisense);

IL-10∶5′-AGAAGCTGAAGACCCTCTGGATAC-3′ (sense),

5′-GCTCCACTGCCTTGCTTTTATT-3′ (antisense);

GAPDH:5′-ATGATTCTACCCACGGCAAG-3′(sense), 5′-CTGGAAGATGGTGATGGGTT-3′(antisense).

The relative levels of IL-1β, IL-10 and MCP-1 mRNAs in each sample were normalized to that of GAPDH levels.

## Supporting Information

Figure S1 **Unchanged Shp1 expression in Shp2 knockdown RBL-2H3 cells.** WT RBL cells were infected with viral supernatants containing recombinant viruses expressing Shp2-specific shRNAs (sh-Shp2-#1 and sh-Shp2-#2) or scramble shRNA (sh-Ctrl), respectively. Puromycin-resistant clones pooled after 5–7 days of infection were harvested to examine Shp2 expression by immunoblotting with anti-Shp1 antibody. Representative blots of three independent experiments are shown.
(TIF)

Figure S2 **Impaired PLCγ1 phosphorylaiton following FcεRΙ aggregation in Shp2 knockdown RBL-2H3 cells.** Indicated populations of RBL cells were sensitized, starved, and stimulated with 10 ng/ml DNP for indicated times. Lysates were immunoprecipitated with PLCγ1 antibody followed by immunoblotting with 4G10 antibody. Representative blots of three independent experiments are shown.
(TIF)
