# Supplementary figures and images for: Shp2 Activates Fyn and Ras to Regulate RBL-2H3 Mast Cell Activation following FcεRI Aggregation
Source: PLoS One. 2012 Jul 10;7(7):e40566. doi: 10.1371/journal.pone.0040566 (PMC3393662; doi:10.1371/journal.pone.0040566)

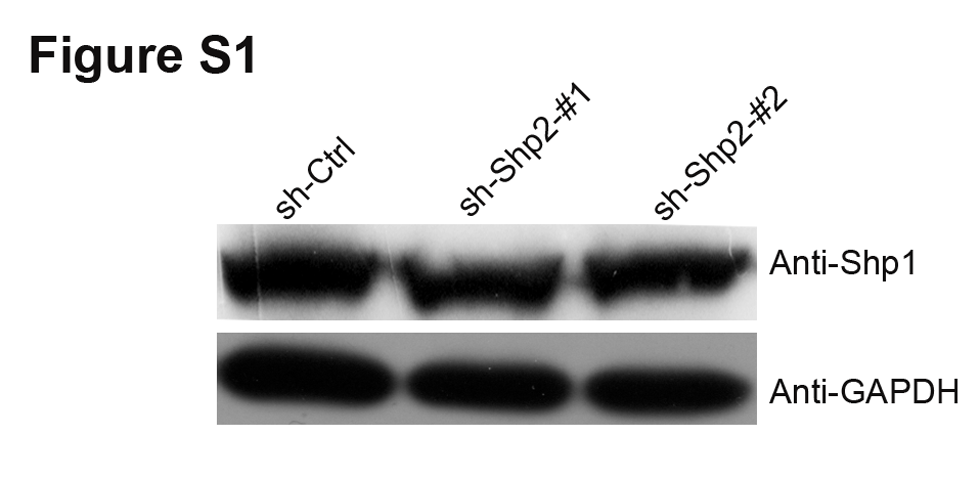

Supplement: Figure S1 — Unchanged Shp1 expression in Shp2 knockdown RBL-2H3 cells. WT RBL cells were infected with viral supernatants containing recombinant viruses expressing Shp2-specific shRNAs (sh-Shp2-#1 and sh-Shp2-#2) or scramble shRNA (sh-Ctrl), respectively. Puromycin-resistant clones pooled after 5–7 days of infection were harvested to examine Shp2 expression by immunoblotting with anti-Shp1 antibody. Representative blots of three independent experiments are shown. (TIF) [file pone.0040566.s001.tif]

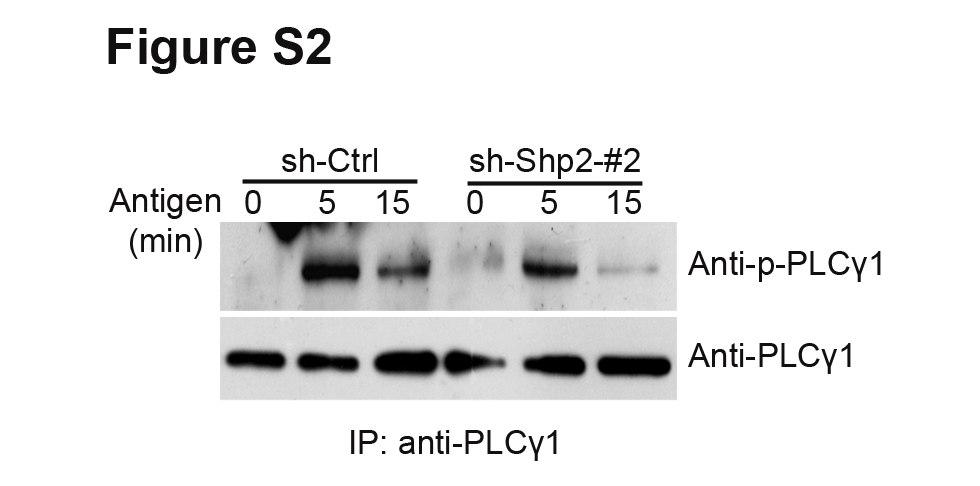

Supplement: Figure S2 — Impaired PLCγ1 phosphorylaiton following FcεRΙ aggregation in Shp2 knockdown RBL-2H3 cells. Indicated populations of RBL cells were sensitized, starved, and stimulated with 10 ng/ml DNP for indicated times. Lysates were immunoprecipitated with PLCγ1 antibody followed by immunoblotting with 4G10 antibody. Representative blots of three independent experiments are shown. (TIF) [file pone.0040566.s002.tif]
